# We Asked the Experts: Surgical Ergonomics: Stop Suffering in Silence

**DOI:** 10.1007/s00268-021-06249-3

**Published:** 2021-07-31

**Authors:** Barbara C. S. Hamilton, Tom C. Nguyen

**Affiliations:** 1 Cardiothoracic Surgery Resident, Division of Cardiothoracic Surgery, Department of Surgery, UCSF, San Francisco, USA; 2 Chief of Adult Cardiothoracic Surgery, Division of Cardiothoracic Surgery, Department of Surgery, UCSF, San Francisco, USA

As surgeons, we have spent years training to excel in a variety of settings: technical masters in the operating room, well-honed clinicians in the wards and clinic, able to function efficiently despite sleep deprivation and without regular meals or breaks. We expect perfection from ourselves; and yet our ability to see the long game regarding our capability to function perfectly is flawed.

Operating, as it turns out, is not just about perfect stitches and precise tension. Over years of training, and with endless nudges from our mentors, we have learned to develop a keen awareness of all activity in the OR, with one exception. While we are acutely tuned in to the field in front of us, the movements of our assistant, the beep of the oxygen saturation monitor, the clock, the dialogue between the anesthesiologist, the perfusionist, and the nurses, there is a key component that we not only forget about but that we may often actively tune out. Only when the skin is being closed, the dressings applied, the bed rolled in, only then do we realize the tension in our necks and backs, the numbness of our laparoscopic thumbs, or the heaviness of the headlight on our heads. Eventually the repetitive movements, the weight, the angles—these take a toll.

We know this instinctively and anecdotally. We see this in our colleagues. We know the classic stance of the senior surgeon with their hunched posture, we know of those who retire early due to neck or back pain. And we know this ourselves. When queried specifically about work-related musculoskeletal pain, 12-month prevalence among surgical specialties is high, with overall estimates of neck, shoulder, and back pain reported at 60%, 52%, and 49%, respectively. [1] While the ergonomics of open surgery have long been recognized as “a mess,” [2] minimally invasive surgical techniques have proven to be even worse, in part given long intervals spent in a static posture. [3] Survey results of minimally invasive surgeons found 86.9% reported physical symptoms or discomfort from operating. [4]

We know our operative ergonomics are a problem. However, it is not in our culture to admit weakness, to confess to our own pain or suffering. In one survey study, [5] of 104 surgeons who sustained a musculoskeletal injury at work, 49% required medical treatment but only 19% of these injuries were reported by the surgeon to their institution. Thirty-five percent of these injuries resulted in a decrease in operative volume, and 54% of injured surgeons felt that these injuries impacted their surgical performance. The ergonomic issue is therefore not only impacting surgeons. Patients, first and foremost, will benefit from surgeons who are able to operate efficiently and comfortably over long careers. Hospitals will benefit from surgeons who are able to maximize their productivity and volume.

The key to ergonomics while operating is relatively simple. The following key components can be addressed to maximize time spent with a neutral body posture while minimizing added weight, while also properly preparing our bodies for the operating room. Think of this as your own prescription for ergonomic success.

Personal equipment considerations:

- Minimizing weight: appropriate selection of headlights, loupes, and lead.
- Loupe declination angle: customized to find the right balance between minimizing the neck angle while avoiding eye strain.
  Operating room set-up:
- Monitor positioning: monitors should be directly facing the surgeon, approximately 1 m away with a declination angle of 0–15 degrees (between the eye and hand level). [6]
- Table height: low enough such that our elbows operate in the neutral position, between 90 and 120 degrees of extension. Optimize complete foot balance by avoiding the use of stepping stools.
- Optimal lighting and high-definition monitors to reduce eye strain.

Operating room conduct:

- Joint positioning: natural upright posture with joint flexion less than half of the available movement of the joint.
- Avoid static posture: every 30–40 min take a 30–60 s break for a few shoulder or neck rolls.

Postoperative recovery and preoperative preparation:

- Daily routines of stretching and resistance can rehabilitate and protect from injury. This can be accomplished in 10 to 15 min and focus on the core, glutes, upper extremities, spine, and neck. Examples include:
  o Core strengthening: mountain climbers, hollow-body hold, planks.
  o Glute strengthening: glute bridge, squats, lunges.
  o Upper extremity stretches: trapezius stretch, levator scapulae stretch, cross-body shoulder stretch.
  o Spine stretch: cat-cow, seated spinal twist.
  o Neck stretch: chin tucks, sternocleidomastoid stretch.
- Consider a daily short yoga routine. A simple sun-salutation sequence will complete a full body stretch while strengthening your core, shoulders, and back. Multiple online platforms exist that can provide guided practice with incorporation into the start or end of your day.

The above considerations need not happen all at once. For the authors, an awareness of ergonomics resulted in definable changes in our practice, leading to equipment adjustments (loupes with an improved angle of declination and attention to monitor heights) and changes in daily routines (10-min yoga practice and a mat in the call-room). These small changes have personally resulted in less neck pain and improved physical recovery following long cases.

Surgical ergonomics is a clear problem with definable consequences. Our ability to operate and to keep operating for years to come is absolutely crucial to our career and our identity as surgeons, not to mention the impact it has on our patients, hospitals, and health care systems. Despite this awareness, proper ergonomics education during surgical training is rarely provided in a consistent fashion. Athletes have a multitude of coaches, physical therapists and trainers ensuring they maintain the ability to function at peak physical performance when it counts, and to obtain career longevity. It is time to speak up, to pay attention to our own ergonomics, and to train ourselves and those we teach in the subtle yet vital art of operative posture. How we position ourselves today will have an enormous impact on our ability to continue to do what we love to do most: operate.

## References

[1] Epstein S, Sparer EH, Tran BN (2018). Prevalence of work-related musculoskeletal disorders among surgeons and interventionalists: a systematic review and meta-analysis. JAMA Surg.

[2] Dudley HA (1977). Micro-ergonomics. Nurs Mirror Midwives J.

[3] Szeto GPY, Cheng SWK, Poon JTC, Ting ACW, Tsang RCC, Ho P (2012). Surgeons' static posture and movement repetitions in open and laparoscopic surgery. J Surg Res.

[4] Park A, Lee G, Seagull FJ, Meenaghan N, Dexter D (2010). Patients benefit while surgeons suffer: an impending epidemic. J Am Coll Surg.

[5] Davis WT, Fletcher SA, Guillamondegui OD (2014). Musculoskeletal occupational injury among surgeons: effects for patients, providers, and institutions. J Surg Res.

[6] Kelts GI, McMains KC, Chen PG, Weitzel EK (2015). Monitor height ergonomics: A comparison of operating room video display terminals. Allergy Rhinol (Providence).

